# [Retracted] Sanmiao formula inhibits chondrocyte apoptosis and cartilage matrix degradation in a rat model of osteoarthritis

**DOI:** 10.3892/etm.2026.13107

**Published:** 2026-02-16

**Authors:** Ying Xu, Guo-Jing Dai, Qian Liu, Zhen-Li Liu, Zhi-Qian Song, Li Li, Wei-Heng Chen, Na Lin

Exp Ther Med 8:1065–1074, 2014; DOI: 10.3892/etm.2014.1862

Following the publication of the above article, a concerned reader drew to the Editor's attention that there were anomalies associated with the photomicrographs shown in Figs. 1 and 3; essentially, groupings of cells appeared to be markedly similar in appearance looking within various of the data panels in these figures.

After having conducted an internal investigation of the data in this paper, the Editor of *Experimental and Therapeutic Medicine* has judged that the potentially anomalous presentation of the strikingly similar groupings of cells in Figs. 1 and 3 were too extensive that these features could have been attributed to pure coincidence. Therefore, the Editor has decided that this article should be retracted from the publication on the grounds of an overall lack of confidence in the data. The authors were asked for an explanation to account for these concerns, but the Editorial Office did not receive a reply. The Editor sincerely apologizes to the readership for any incovenience caused, and we thank the reader for bringing this matter to our attention.

